# Prevalence and Associated Factors of Diabetes Mellitus in Hosanna Town, Southern Ethiopia

**DOI:** 10.5334/aogh.2663

**Published:** 2020-02-24

**Authors:** Nebiyu Dereje, Alemu Earsido, Layla Temam, Ashenafi Abebe

**Affiliations:** 1 Department of Public Health, Wachemo University, Hosanna, ET; 2 Department of Medicine, Wachemo University, Hosanna, ET; 3 Department of Statistics, Wachemo University, Hosanna, ET

## Abstract

**Background/Objectives:**

Diabetes is a global public health problem, and its burden is rising, particularly in developing countries. However, limited data is available from sub-Sahara African communities to assess and monitor the disease burden. The study aimed to determine the prevalence and associated factors of diabetes in Hosanna, Ethiopia.

**Methods:**

A community-based cross-sectional study was conducted among 634 randomly selected adults in Hosanna. The study participants were recruited by multi-stage stratified sampling. A face-to-face interview using a structured questionnaire was administered by trained nurses. Anthropometry, blood pressure and fasting blood glucose levels were measured. Diabetes mellitus was considered when the fasting blood glucose level was ≥126 mg/dl on two separate measurements or when the participant self-reported a previous diagnosis of diabetes by healthcare providers or when the participant was currently receiving treatment for diabetes. Multi-variable binary logistic regression was used to identify factors associated with diabetes mellitus.

**Findings:**

The overall prevalence of diabetes was found to be 5.7% (95% CI; 4.0–7.7), out of which more than one third (36%) were not aware of it prior to the survey. Nearly two thirds (61.1%) of the diabetic participants were also found to be hypertensive. In the multi-variable analysis, diabetes was associated with current alcohol use, sitting on average of more than 8 hours/day, abnormal BMI and being hypertensive.

**Conclusion:**

The prevalence of diabetes among the adult population in the town is alarming. If appropriate measures to address the burden are not emplaced, it might result in serious complications to the patients and unnecessarily high costs to the health system of the country. Active screening for raised blood glucose level should be given due consideration, particularly in the community setting. Designing health education programs on the importance of physical activity and the risks of alcohol use should also be considered.

## Introduction

Diabetes mellitus is a chronic disease of elevated blood glucose level due to either suboptimal production of insulin by the pancreas or peripheral resistance of the body to insulin [1]. It is one of the leading global health problems, accountable for about half a billion cases and 1.6 million deaths of the adult population in 2016 [2,3,4]. Despite efforts by the World Health Organization (WHO) to reduce the burden of diabetes, its prevalence is increasing and might cause further premature death and an estimated economic burden of $2.1 trillion (2.2% share of the global GDP) in 2030 [2].

Although, the prevalence of diabetes in Sub-Sahara Africa was reported to be lower (3%) as compared to the global prevalence (8.5%) in 2016, there is a rising trend in the past few decades due to the effects of rapid urbanization, globalization and life style changes [5]. Moreover, it is alarming that more than two thirds of the diabetes mellitus cases were undiagnosed in the Sub-Sahara Africa [3,4,6,7,8,9].

In Ethiopia, according to the national WHO STEPS survey of 2015, the prevalence of diabetes mellitus was 3.2% [10]. A few other studies in Ethiopia report the prevalence of diabetes mellitus in a range from 0.5% to 6.5% [11,12,13,14,15,16,17]. Moreover, the prevalence of undiagnosed diabetes, those who are neither aware of raised blood sugar nor taking any anti-diabetic medications, was very high in Ethiopia [7,8,9,18]. If diabetes is not recognized early and treated, it may pose serious health problems as it progresses to affect other organs of the body [7,19,20]. However, the prevalence of diabetes (diagnosed and undiagnosed) among the adult population of Hosanna is unknown. Not only the prevalence but also factors associated with diabetes mellitus are not known in the town, and previously conducted studies in other parts of Ethiopia have reported inconsistent and inconclusive findings with regard to risk factors of diabetes mellitus [13,14,15,16]. These studies reported older age, a family history of diabetes, hypertension and physical inactivity are associated with diabetes. However, none of the prior studies reported on dietary factors, abnormal BMI and behavioral factors. There is no evidence to direct the decision-making abilities of the health system policy makers, programs and actors, which in turn has an implication on budget allocation and resource distribution. There has been much investment to prevent and control communicable diseases in the town, but appropriate attention has not been given yet for the control of non-communicable diseases, including diabetes. Therefore, this study aims to assess the prevalence and associated factors of diabetes mellitus and has generated evidence that will benefit the people of the town in several ways. For instance, the findings can be used by the health system as a baseline for planning, budgeting and resource allocation. The findings can also be used by researchers for advocating healthy life styles and by programmers to design appropriate, effective and efficient interventions to prevent and control diabetes mellitus.

## Methods and Materials

### Study setting and population

Data for this study were obtained from a community-based cross-sectional study conducted from May 15 to May 20, 2017, among a selected adult population (≥18 years) in Hosanna, Ethiopia. The town is structured in 3 sub-cities, having 12 kebeles (the lowest administrative unit in Ethiopia). According to the 2007 national census report, the town had a population of 69,995.

### Sample size and sampling procedures

A sample of 634 was estimated using the single population proportion formula by taking 95% confidence interval (CI), 5% margin of error, design effect of 2, 5.1% prevalence of diabetes mellitus in North West Ethiopia [13] and adding up a 10% non-response rate. Multi-stage stratified sampling was employed to recruit the samples. In the first stage, out of the three sub-cities of the town, three kebeles from each were randomly selected by lottery (total of nine kebeles). The number of households to be included from each kebele was proportional to the population. Then, households to be included in the study were selected by simple random sampling (computer-based random number generator) using the health extension workers family folder and registry as a sampling frame. In the Ethiopian health system, the lowest health facility located near the community is called a health post. In this level of health facility, the health extension workers provide basic services on immunization, family planning, antenatal care, health education, nutritional supplements and some level of treatment for different disease conditions. To facilitate these, the health extension workers have a family folder for each household (family) in their catchment. Each family folder contains the address of the household, the list of individuals in that family and their age groups and different household characteristics (e.g., type of latrine they have). The health extension workers follow the population in their catchment based on the family folder and update any vital events, such as birth and death. Moreover, the health extension workers have the overall list (registry) of their population in their catchment area. The research team used this list of individuals as a sampling frame to select participants for the study. All the eligible individuals in the selected households were included in the study. Individuals who were critically ill and unable to respond for the interview were excluded from the study.

### Data collection tools and procedures

Face-to-face interviews were conducted by trained nurses using a structured questionnaire adapted from the WHO STEPwise approach to chronic disease surveillance tool [21]. Information on tobacco use, alcohol consumption, fruit and vegetable consumption, physical activity, physical measurement, blood glucose level, chronic disease history and family health was collected. Standard procedures adapted from the WHO STEPwise guideline were used to measure weight, height, blood pressure and blood glucose level. Height was measured by sliding meter and read in centimeters to the nearest 0.1 cm and recorded. Weight was measured using a digital weighing scale (UNICEF seca) and recorded in kilograms to the nearest 0.1 kg. Blood pressure measurements were taken using a digital blood pressure measuring apparatus (digital sphygmomanometer). Two measurements of blood pressure for each participant were taken for analysis purposes, recording the mean of the first and second readings. The right arm was used for this measurement. The displayed reading of the systolic and diastolic blood pressure was recorded. Participants rested for ten minutes between each reading [21].

The research investigators were responsible for the overall management of the project: for development of the final questionnaire, for making the initial contact with and securing participation of the kebeles selected, for identifying survey administrators and for training and assigning them to the selected kebeles. Data collectors were 15 trained nurses and public health professionals who were working in the study area at the time of data collection and who were able to communicate with the local language.

On the first day, interviews were held at the participants’ homes. Eligible participants were declared unavailable if they were not found on three separate visits. In six households, adult participants were unavailable on repeated visits during the data collection period and were replaced by the next eligible households. On the subsequent day, anthropometric, blood pressure and fasting blood glucose measurements were taken at health centers nearer to the community. A blood sample for measuring the fasting blood glucose level was taken after the participants of the study fasted overnight for at least 12 hours [1,8]. Blood glucose level was measured using a glucometer. Seven participants were not able to visit the health center for anthropometric, blood pressure and fasting blood glucose measurements, and they were excluded from the analysis.

All study instruments were translated into the local language (Amharic) by native speakers and then back translated to English by other persons who understand the languages to check consistency. Pretesting was conducted in 5% of the samples to see the completeness, consistency and applicability of the instruments and was ratified accordingly. Instruments used for measuring the physical dimensions, such as the digital weighing scale and height measuring board, were calibrated on a daily basis and checked after every measurement.

Daily supervision was made in the field by field supervisors and investigators during data collection. Data collectors checked for data completeness and consistency before leaving each house. Field supervisors also checked the completeness and consistency of the data on a daily basis, and they returned to interviewers if the data were incomplete and inconsistent.

### Measurement of variables

Diabetes mellitus (raised blood glucose level) was defined as fasting blood glucose level ≥126 mg/dl on two separate measurements or a self-report of previous diagnosis by healthcare providers or receiving treatment for diabetes. Undiagnostic diabetes was defined as participants whose fasting blood glucose level was ≥126 mg/dl on two separate measurements and were unaware of it prior to the survey [1,13,15].

Raised blood pressure (hypertension) was defined as a systolic blood pressure ≥90 mmHg or diastolic blood pressure ≥140 mmHg on two separate measurements or taking anti-hypertensive drugs to control their raised blood pressure [22,23,24,25].

Current smoking and current alcohol use was defined as using tobacco products and alcohol within the preceding month prior to the survey [21].

Weight divided by height squared (KG/M^2^) was used to compute the body mass index (BMI). BMI was categorized as normal if it was <25 KG/M^2^; overweight, if it was from 25–29.9 KG/M^2^ and obese if it was ≥30 KG/M^2^. However, for the analysis, we categorized BMI into two groups: normal and abnormal (merging the overweight and obese group) [26,27,28].

Physical activity was measured by using the WHO physical activity questionnaire [21]. The questionnaire assessed work-related activity, walking, sport and recreational activity, and time spent sitting per day. Work-related activities were categorized as work involving vigorous intensity and moderate intensity activities. Work involving vigorous intensity activity was measured by asking the question ‘Does your work involve vigorous-intensity activity that causes large increases in breathing or heart rate like (*carrying or lifting heavy loads, digging or construction work*) for at least 10 minutes continuously?’, and work involving moderate intensity activity was measured by asking the question ‘Does your work involve moderate-intensity activity that causes small increases in breathing or heart rate such as brisk walking (*or carrying light loads*) for at least 10 minutes continuously?’ Based on this, an adult person should do at least 150 minutes of moderate intensity work or 75 minutes of vigorous intensity work or 60 minutes of a combination vigorous and moderate intensity work per week. If the self-reported physical activity did not fit with the WHO recommendation, participants were categorized as physically inactive [21,26,27,28,29].

### Data management and analysis

Data were checked, cleaned and entered into Epi data 3.1, then imported into SPSS version 20 for analysis. Seven participants’ data were found to be incomplete and thus excluded from the analysis. Descriptive statistics were used to summarize the data. The prevalence of diabetes mellitus was described using proportion and 95% CI. Associations between independent variables (age, sex, cigarette smoking, alcohol use, physical inactivity, etc.) and diabetes mellitus were analyzed first using bivariate analysis (in the binary logistic regression) to identify factors eligible for the multivariable binary logistic regression analysis. Those variables with *p*-value < 0.25 in the bivariate analysis were included in the multi-variable analysis. Magnitude of the association between the independent and dependent variables were measured using the odds ratios (OR) and 95% CI, and P values below 0.05 were considered statistically significant. Multi-collinearity between variables was assessed using the multi-collinearity diagnostics (Variance Inflation Factor and tolerance test). The final multi-variable binary logistic regression model was found to fit based on the finding of the Hosmer-Lemeshow goodness-of-fit test.

### Ethical considerations

Before beginning data collection, ethical clearance was obtained from the Wachemo University institutional review board (WCU/IRB/086/17) and informed written consent of the participants was obtained during the data collection. Those participants with an elevated fasting blood glucose level and blood pressure were linked to the health centers for appropriate care and treatment.

## Results

### Participants’ socio-demographic characteristics

As presented in Table 1, among the total participants (n = 627), 58.5% were males and 47.2% were above 35 years. About two thirds (65.6%) of the participants were married, and about one third were self-employed (31.6%).

**Table 1:** Socio-demographic characteristics of the study participants in Hosanna, 2017 (n = 627).

Variable	Response	Frequency (percent)

**Sex**	Female	260 (41.5%)
	Male	367 (58.5%)
**Age**	≤35 years	331 (52.8%)
	>35 years	296 (47.2%)
**Marital status**	Single	216 (34.4%)
	Married	411 (65.6%)
**Educational status**	Illiterate	32 (5.1%)
	Literate	595 (94.9%)
**Occupation**	Government/NGO employed	219 (34.9%)
	Self employed	198 (31.6%)
	Student	59 (9.5%)
	Housewife	87 (13.9%)
	Retired	21 (3.3%)
	Unemployed	43 (5.8%)

### Prevalence of Diabetes Mellitus

The overall prevalence of diabetes mellitus was found to be 5.7% (95% CI; 4.0–7.7), 6.5% among the males and 4.6% among the females. Among the diabetics, only about two thirds were told that they were diabetic by the health professionals, and the remaining one third (36%) were not aware of it before. Those participants who were aware of their diabetic status were taking different measures to control the disease, such as taking insulin (65.2%), taking oral anti-glycemic medications (34.8%), dietary modifications (73.9%), traditional medicine (39.1%) and exercise (39.1%). As indicated in Table 2, the prevalence of diabetes was found to be highest among hypertensive participants.

**Table 2:** Prevalence of diabetes mellitus among adult population in Hosanna, 2017.

Prevalence of diabetes mellitus	Percent	95% CI

Overall (n = 627)	5.7	4.0–7.7
Among current smokers (n = 30)	6.7	0.1–17.4
Among current alcohol users (n = 100)	13.0	6.5–20.0
Among physically inactive participants (n = 107)	5.6	1.8–10.4
Among those who didn’t consume fruit (n = 125)	7.2	3.0–12.0
Among those who didn’t consume vegetables (n = 56)	8.9	1.8–17.9
Among those who commonly use saturated or unspecified oil (n = 549)	6.0	4.2–8.0
Among those with abnormal BMI (n = 153)	10.5	5.9–15.9
Among hypertensive participants (n = 108)	20.4	13.1–28.3

### Participants’ behavioral characteristics

Overall, 4.8% of the participants reported they were current cigarette smokers (within the preceding month). Whereas, 7.2% of the participants ever smoked cigarettes in their lifetime. Among the current and ever smokers, only about 7% and 8.8%, respectively, were found to be diabetic.

The prevalence of current alcohol use among the study participants was 15.9%. A substantial proportion of the alcohol users were in the age group of 35–44 years. Among the current alcohol users, 13% were found to be diabetic.

In the bivariate analysis (Table 3), current alcohol use has a statistically significant association with diabetes. However, current cigarette smoking was not significantly associated with diabetes.

**Table 3:** Socio-demographic and behavioral characteristics of the participants and the association with diabetes mellitus in Hosanna, 2017.

Variables	Diabetes Mellitus		COR	95% CI	*P* value

	Yes	No			

**Sex**					
Female	12 (33.3%)	284 (42.0%)	1.45	0.71–2.95	0.31
Male	24 (66.7%)	343 (58.0%)	1.00		
**Age**					
<35 years	13 (36.1%)	318 (53.8%)	1.00	1.02–4.15	0.04*
≥35 years	23 (63.9%)	273 (46.2%)	2.06		
**Educational status**					
Literate	33 (91.7%)	562 (95.1%)	1.00		
Illiterate	3 (8.3%)	29 (4.9%)	1.76	0.51–6.01	0.37
**Current smoker**					
Yes	2 (5.6%)	28 (4.7%)	1.2	0.27–5.17	0.82
No	34 (94.4%)	563 (95.3%)	1.00		
**Current alcohol use**					
Yes	13 (36.1%)	87 (14.7%)	3.30	1.60–6.70	0.001*
No	23 (63.9%)	504 (85.3%)	1.00		

* Significantly associated.

### Participants’ dietary patterns and physical activity

In the week prior to the survey, about one out of five and one out of ten participants, respectively, had not consumed fruits and vegetables. Among those who had consumed fruits and vegetables, about 78% and 90%, respectively, consumed less than five servings of fruits and vegetables per day. Only 7.2% and 8.9%, respectively, of those who had not consumed fruits and vegetables were found to be diabetic.

A considerable proportion of the participants (87.5%) reported they have commonly used saturated fats (oils) or unspecified different types of fats (oils) for meal preparation; out of them, 6% were diabetic.

In the week prior to the survey, 30% of the participants ate meals outside of their home more than two times. Out of those, 5.9% were diabetic. In the bivariate analysis (Table 4), none of the dietary factors showed a statistically significant association.

**Table 4:** Dietary and physical activity related factors of the participants and the association with diabetes mellitus in Hosanna, 2017.

Variables	Diabetes Mellitus		COR	95% CI	*P* value

	Yes	No			

**Physical Activity**					
Not meet WHO recommendation	30 (83.3%)	490 (82.9%)	1.03	0.42–2.54	0.95
Meet WHO recommendation	6 (16.7%)	101 (17.1%)	1.00		
**Fruit consumed in the last week**						
Yes	27 (75.0%)	475 (80.4%)	1.00		
No	9 (25.0%)	116 (19.6%)	1.37	0.63–2.98	0.44
**Vegetables consumed in the last week**						
Yes	31 (86.1%)	540 (91.4%)	1.00		
No	5 (13.9%)	51 (8.6%)	1.71	0.64–4.58	0.29
**Types of oil commonly used**						
Unsaturated	3 (8.3%)	75 (12.7%)	1.00		
Saturated or unspecified	33 (91.7%)	516 (87.3%)	1.60	0.48–5.34	0.45
**BMI**						
Normal	20 (55.6%)	454 (76.8)	1.00		
Abnormal	16 (44.4%)	137 (23.2)	2.65	1.34–5.26	0.005*
**Time spent sitting in the last week**						
1–4 hours/day	13 (36.0%)	196 (33.2%)	1.00		
5–8 hours/day	16 (44.4%)	343 (58.0%)	2.00	0.70–5.40	0.15
>8 hours/day	7 (19.4%)	52 (8.8%)	2.89	1.13–7.35	0.026*
**Hypertensive**						
Yes	22 (61.1%)	86 (14.6%)	9.23	4.55–18.73	0.0001*
No	14 (38.9%)	505 (85.4%)	1.00		

* Significantly associated.

According to the WHO recommendation, an adult person should do at least 150 minutes of moderate intensity work or 75 minutes of vigorous intensity work or 60 minutes of a combination of vigorous and moderate intensity work per week [28]. However, only 17.1% of the respondents’ physical activity met the WHO recommendations. Moreover, 7.8% of the participants did not walk or bicycle in the week prior to the survey. In addition, about 9.4% of the respondents spent an average of more than 8 hours per day sitting.

In the bivariate analysis (Table 4), physical activity of the participants was not significantly associated with diabetes mellitus. However, those who spent an average of more than 8 hours per day sitting had an increased risk of diabetes.

### Participants’ physical and blood pressure measurements

The mean weight, height and BMI of the participants were 66.98 ± 8.1 KG, 1.69 ± 0.1 M and 23.6 ± 3.4 KG/M^2^, respectively. About one quarter (24.4%) of the participants had abnormal BMI (either overweight or obese). The prevalence of diabetes mellitus among the overweight or obese participants was found to be 10.2%. Abnormal BMI showed a statistically significant association with diabetes (Table 4).

Blood pressure was measured two times and the average was recorded. The mean systolic blood pressure was 123.4 mmHg, and the mean diastolic blood pressure was 75.3 mmHg. With regard to their hypertensive status (defined as systolic blood pressure of greater than or equal to 140 mmHg and/or diastolic blood pressure of greater than or equal 90 mmHg), 17.2% were found to be hypertensive.

### Factors associated with diabetes mellitus

In the bivariate analysis, variables significantly associated with diabetes mellitus were age ≥ 35 years, current alcohol use, spending on average more than 8 hours per day sitting, abnormal BMI and hypertension. Further multi-variable analysis was performed to control the effects of confounders and to estimate the independent effects of the explanatory variables on the outcome variable. Accordingly, current alcohol use, sitting on average more than 8 hours per day, abnormal BMI and hypertension were found to be significantly associated with diabetes mellitus (Table 5).

**Table 5:** Factors associated with diabetes mellitus among adult population in Hosanna, 2017.

Variables	Diabetes Mellitus		COR (95% CI)	AOR (95% CI)	*P*-value

	Yes	No			

**Age**					
<35 years	13	318	1.00	1.00	
≥35 years	23	273	2.06 (1.02–4.15)	1.52 (0.72–3.21)	0.23
**Current alcohol use**					
Yes	13	87	3.30 (1.60–6.70)	3.31 (1.58–6.92)	0.002*
No	23	504	1.00	1.00	
**BMI**					
Normal	20	454	1.00	1.00	
Abnormal	16	137	2.65 (1.34–5.26)	2.12 (1.04–4.33)	0.04*
**Time spent sitting in the last week**					
1–4 hours/day	13	196	1.00		
5–8 hours/day	16	343	2.00 (0.70–5.40)	1.86 (0.65–5.32)	0.25
8 hours/day	7	52	2.89 (1.13–7.35)	2.48 (1.05–6.59)	0.04*
**Hypertensive**					
Yes	22	86	9.23 (4.55–18.73)	7.36 (3.35–16.17)	0.0001*
No	14	505	1.00		

* Significantly associated.

The odds of developing diabetes mellitus was three times more likely among those who currently use alcohol than their counterparts (AOR = 3.31, 95% CI; 1.58–6.92). The odds of developing diabetes mellitus among those who were sitting on average more than 8 hours per day was 2.5 times higher than those who sat less than 4 hours per day (AOR = 2.48, 95% CI; 1.05–6.59). The odds of developing diabetes mellitus was two times higher among those with abnormal BMI compared to those with normal BMI (AOR = 2.12, 95% CI; 1.04–4.33). Similarly, those participants who were hypertensive were seven times more likely to be diabetic than those who were not hypertensive (AOR = 7.36, 95% CI; 3.35–16.17).

## Discussion

The burden of diabetes mellitus and its associated modifiable risk factors are increasing in developing countries like Ethiopia [6,30,31]. A community-based cross-sectional study to assess the prevalence and associated factors of diabetes was conducted in Hosanna, Ethiopia. The study uncovered an unacceptably high overall prevalence of both diagnosed and undiagnosed diabetes mellitus (5.7%) among the adult population. Alarmingly, more than one third (36%) of the diabetic population were not aware of their diabetic status prior to the survey. If diabetes mellitus is not timely undiagnosed and left unrecognized, it may cause serious complications, long-term disabilities and pre-mature death [7,9]. This problem in part may reflect a lack of adequate awareness on screening of diabetes among the community members, and in part, it reflects an absence of quality healthcare facilities that mobilize active screening services in the community. This finding calls for active community-based screening programs for early detection of diabetes and emphasizes the need for universal health coverage.

The overall prevalence of diabetes mellitus in Hosanna is much higher than the 3.2% reported nationally by the WHO STEPs survey [10]. However, it is comparable with the prevalence reports from Northwest Ethiopia (4.9% and 5.1%) [13,17] and Addis Ababa (6.5%) [12]. The discrepancies in the prevalence might be because of the fact the WHO STEPs survey included both urban and rural populations. As expected and reported by other studies conducted in Ethiopia, the prevalence of diabetes mellitus in the rural population is lower compared to the urban population. This is due to lifestyle differences among the rural and urban populations [8,13,16,25].

Current alcohol use was found to be associated with diabetes mellitus, which is supported by other studies. [32,33] However, a study conducted in Northwest Ethiopia [13] reported protective effects of alcohol use on diabetes mellitus. The differences on the dosage and patterns of alcohol use might have influenced the findings of the studies. Moreover, the participants of the respective studies may have reported no use or lower use, as alcohol use is not well accepted by Ethiopian. However, the findings of this study revealed an increased risk of diabetes mellitus among those who use alcohol irrespective of the dosage and type of alcohol. This can be explained by the fact chronic alcohol use might cause dysfunction of the pancreatic β – cells and insulin resistance [32,33]. Moreover, alcoholic beverages can directly increase the blood sugar level, stimulate appetite and affect decision making for dietary planning [34,35]. It is to be noted that alcohol use in the town is high (15.9%) and should be actively controlled by creating awareness in the community on the risks of alcohol use.

Physical inactivity (sedentary life) is also identified as a risk factor for the development of diabetes mellitus. This study’s findings are consistent with other studies [26,27,29,36] and supportive of the WHO recommendation of physical activity (doing at least 150 minutes of moderate intensity work or 75 minutes vigorous intensity work or 60 minutes of a combination of vigorous and moderate intensity work per week) [28].

Consistent with other studies [8,26,37], abnormal BMI (obesity) was found to be associated with diabetes mellitus. These findings suggest weight control and physical activity should be considered as important interventions for the prevention and control of diabetes mellitus in the town. Because the prevalence of obesity was found to be high and the practice of physical activity was low, strong commitment and action is demanded to halt the high burden of diabetes mellitus.

In the present study, raised blood pressure (hypertension) was found to be a strong risk factor for the development of diabetes mellitus, which is consistent with other studies [15,18,23,38]. A considerable proportion of the participants had both hypertension and diabetes mellitus. This finding is alarming and suggests measures are necessary to control the twin trajectories. Moreover, what makes this finding serious is that more than one third of the diabetic patients were not aware of their elevated blood pressure and blood sugar prior to the survey. Unless timely measures to control elevated blood pressure are taken, microvascular and macrovascular complications of diabetes mellitus are inevitable and might be worsened. This in turn will escalate the cost of treatment and will have a significant negative impact on the health system of the country.

### Strengths and limitations

The present study employed a community-based design, which allows for generalization to the population of the town. The study employed three data collection methods (participant interviews, physical measurements, and blood tests using standard procedures), which allowed for the inclusion of all potential risk factors (variables) and for triangulating the study findings from different sources. Moreover, the WHO STEPwise approach to chronic diseases surveillance tool was used to conduct this study, which can provide comparability of our findings with other similar studies globally.

However, this study has limitations because it employed a cross-sectional study design and because it was not possible to ascertain the temporal relationships between the variables. In addition to this, some of the variables were taken for a study period only. For instance, nutrition-related questions were assessed for one week prior to the survey and might not represent the usual pattern and participants may have been prone to recall bias. Moreover, alcohol use related variables were not inclusive of the locally available products as they are difficult to measure. Furthermore, as the number of current smokers in our study were few, its statistically significant association with diabetes was masked. Differentiation of Type 1 and Type 2 diabetes mellitus was not possible in this study. Therefore, we recommend research that can address these problems by employing observational study designs and by recruiting study participants prospectively.

## Conclusion

The prevalence of both diagnosed and undiagnosed diabetes mellitus among the adult population in Hosanna is high. If appropriate measures to address the burden are not emplaced, it might result in serious complications to the patients and unnecessary high costs to the health system of the town and the country. The burdens of modifiable risk factors of diabetes (alcohol use, obesity, physical inactivity and raised blood pressure) were also found to be high in the town. Active screening for raised blood sugar levels and blood pressure should be given due consideration, particularly in the community setting. Designing health education programs on the importance of physical activity and the risks of alcohol use are critical to controlling the burden of diabetes mellitus.
